# Impact of Downward Load and Rotational Kinematics on Root Canal Instrumentation with a Heat-Treated Nickel–Titanium Rotary Instrument

**DOI:** 10.3390/ma19010108

**Published:** 2025-12-28

**Authors:** Risako Yamamoto, Keiichiro Maki, Shunsuke Kimura, Satoshi Omori, Keiko Hirano, Arata Ebihara, Yoshio Yahata, Takashi Okiji

**Affiliations:** 1 Department of Pulp Biology and Endodontics, Division of Oral Health Sciences, Graduate School of Medical and Dental Sciences, Institute of Science Tokyo, 1-5-45 Yushima, Bunkyo-ku, Tokyo 113-8549, Japan; r.yamamoto.endo@tmd.ac.jp (R.Y.); s.kimura.endo@tmd.ac.jp (S.K.); s.omori.endo@tmd.ac.jp (S.O.); k.hirano.endo@tmd.ac.jp (K.H.); a.ebihara.endo@tmd.ac.jp (A.E.); yahata.yoshio@tmd.ac.jp (Y.Y.); t.okiji@tky.ndu.ac.jp (T.O.); 2 Department of Endodontics, The Nippon Dental University School of Life Dentistry at Tokyo, 1-9-20 Fujimi, Tokyo 102-8159, Japan

**Keywords:** nickel–titanium rotary instrument, root canal treatment, optimum torque reverse mode, canal-centering ratio, torque, vertical force, downward load, instrumentation time

## Abstract

This study analyzed how different downward loads and rotational kinematics influence NiTi rotary instrumentation outcomes. Heat-treated NiTi instruments were used to prepare extracted human single-rooted premolars with a moderate canal curvature. Instrumentation was performed using an automated endodontic instrumentation device with controlled downward loading and torque/force sensing, under different downward load settings (1, 2, and 3 N), employing either continuous rotation (CR) or optimum torque reverse (OTR) motion, which is a torque-sensitive reciprocation. Instrumentation was completed without instrument fracture or ledge formation in all six groups. OTR-3N specimens displayed a significantly lower upward force (i.e., screw-in force) than OTR-2N specimens (*p* < 0.05). OTR-1N specimens required a significantly longer instrumentation time than CR-1N specimens and the other OTR specimens (*p* < 0.05). At 1 mm from the apex, CR-2N specimens showed a significantly larger canal-centering ratio (i.e., larger deviation) than OTR-2N specimens (*p* < 0.05). Overall, applying a downward load of 2–3 N in OTR mode provided shaping efficiency similar to CR, but with a reduced screw-in force and enhanced canal-centering in the apical region, supporting the use of OTR as a promising alternative to CR for curved canal preparation using heat-treated NiTi instruments.

## 1. Introduction

Apical periodontitis develops as an inflammatory response of the periradicular tissues to bacterial infections originating from the root canal system. Root canal instrumentation plays a critical role in mechanically eliminating infected material, shaping the root canal to facilitate efficient irrigation, and preparing it for proper obturation, thereby reducing the microbial load and promoting healing of the periapical tissues [1,2,3]. Nickel–titanium (NiTi) rotary instruments have become widely accepted for root canal treatments owing to their superior flexibility [4], which helps preserve the original root canal curvature. Nevertheless, clinical challenges persist, such as unexpected instrument fracture [5] and canal transportation [6,7]. To overcome these issues, NiTi rotary instruments have undergone progressive advancements, such as heat treatments that enhance their flexibility [8] and improved cross-sectional design that increases cutting efficiency while minimizing excessive stress during instrumentation [9,10,11].

Beyond improvements in material properties, operational factors, such as insertion speed [12] and the magnitude of the vertical load [13], have a substantial impact on the performance of root canal instrumentation. According to a previous study [13], applying a larger downward load during root canal instrumentation using ProTaper NEXT rotary instruments (Dentsply Sirona, Charlotte, NC, USA) offers several advantages, including reduced apical canal deviation and instrumentation time. However, the study also reported an increase in the screw-in force—a self-propelling upward force that pulls the instrument deeper into the canal, causing sudden engagement with the canal wall and potentially leading to intracanal fracture [9,14]. These findings indicate that, although larger downward loads may improve shaping outcomes when torque generation is appropriately controlled, careful management of screw-in forces remains essential. Those earlier studies assessed the influence of operational factors in simulated resin canals, but the differences in their material properties compared with natural root canal dentin present limitations [15].

The rotation mode, defined as the specific kinematic motion applied by NiTi rotary instruments, is another critical factor influencing the quality of root canal treatment outcomes. Two principal kinematic modes are currently employed in NiTi instrumentation: continuous rotation (CR), in which the instrument rotates 360° clockwise, and reciprocation, in which the instrument alternates between clockwise and counterclockwise directions [16,17]. The optimum torque reverse (OTR) mode is a torque-sensitive reciprocating motion that automatically switches from CR to reciprocation when a preset torque limit is exceeded [18]. This adaptive function has been successfully applied in clinical practice, providing reductions in both the torque and screw-in force [19], enhanced cyclic fatigue resistance [20,21], and improved canal-centering ability [18].

Operators can freely select between rotational modes (e.g., CR and OTR), but the optimum instrument manipulation, especially the magnitude of the downward load, may vary with the selected rotational mode. Notably, evidence regarding the interaction between downward load magnitude and rotational mode remains insufficient, and to our knowledge no previous studies have systematically investigated this relationship using extracted human teeth.

Therefore, the present study examined how varying downward loads and rotational modes (CR and OTR) influence NiTi rotary instrumentation, specifically focusing on torque generation, vertical force development, instrumentation time, and canal-centering ability. By using extracted human teeth rather than simulated resin canals, this study sought to provide clinically relevant insights for optimizing instrument manipulation. The primary outcome was the canal-centering ratio, an indicator of root canal shaping quality. The secondary outcomes included instrumentation time, maximum torque and vertical force values, and the incidence of procedural complications such as instrument fracture and ledging. The null hypothesis stated that neither downward load nor rotational mode significantly affects instrumentation outcomes.

## 2. Materials and Methods

### 2.1. Sample Size Calculation

The number of specimens required for this study was calculated using G*Power software (version 3.1.9.7; Heinrich Heine Universität, Düsseldorf, Germany). An a priori one-way ANOVA (fixed effects, omnibus) was applied. The effect size (f = 0.6) was estimated based on the between-group differences in maximum screw-in force observed in our preliminary experiments under the same experimental conditions. Assuming a significance level (α) of 0.05 and a statistical power of 0.85, a minimum of nine specimens per group was established and implemented in this study.

### 2.2. Tooth Selection

Ninety mandibular premolars extracted for clinical indications unrelated to the study were obtained under Institutional Review Board approval (Tokyo Medical and Dental University, currently Institute of Science Tokyo; no. D2014-033).

Using periapical digital radiographs, teeth with caries, fractures, restorations, an immature apex, or resorption were excluded. The remaining specimens were then decoronated using a diamond bur (ISO 6360-1:2004, size 173, diameter code 014 (MANI, Utsunomiya, Japan) [22].

To evaluate the canal configuration, micro-computed tomography (micro-CT; inspeXio SMX-100CT; Shimadzu, Kyoto, Japan) was performed at 70 kV and 100 mA, with a 1-mm aluminum filter, employing a 360° rotation around the vertical axis in 0.5° rotation steps, and frame averaging of 5, producing images with a voxel size of 0.03 mm. The teeth were secured by pouring self-curing resin (Unifast III, GC, Tokyo, Japan) into a customized jig for fixation to the micro-CT apparatus. Then, images for each tooth were analyzed using three-dimensional visualization and analysis software (Amira v. 2023.2; Thermo Fisher Scientific, Waltham, MA, USA). Ultimately, 54 teeth that met the following criteria were selected: a single oval canal (defined as having a buccolingual dimension approximately 1.5 times greater than the mesiodistal dimension at a level 5 mm from the apex), a moderate curvature ranging from 15° to 25°, based on the Schneider method [23], and measurements of the canal cross-sectional area obtained at the 1-mm and 5-mm levels from the root apex. We selected canals with curvatures of 15–25° because this range closely corresponds to the morphology of transparent resin canals commonly used in previous studies [24], allowing methodological consistency and comparison with established experimental models. Specimens were randomly allocated to six experimental groups (*n* = 9 each) according to the downward load (1, 2, or 3 N) and the operational mode (CR or OTR). No significant differences in geometric parameters were observed among the groups (Table 1), as determined by one-way analysis of variance following confirmation of normality (Kolmogorov–Smirnov and Shapiro–Wilk tests) and homogeneity of variance (Levene’s test).

### 2.3. Automated Endodontic Instrumentation Device with Controlled Downward Loading and Torque/Force Sensing

The Tri-Auto ZX endodontic motor (J Morita, Kyoto, Japan) was operated in either CR or OTR mode, using torque threshold settings as previously reported [25]. For CR mode, the programmed torque limit was set at 3 N·cm, as recommended by the manufacturer for JIZAI instruments (MANI, Utsunomiya, Japan) (≤3 N·cm). In OTR mode, when the preset torque limit of 0.8 N·cm—corresponding to the default OTR activation value—was exceeded, the motion switched from CR to a reciprocating motion consisting of 90° counterclockwise and 180° clockwise rotations. A rotational speed of 500 rpm was employed in accordance with the manufacturer’s recommended settings. In OTR mode, although the nominal speed was set at 500 rpm, effective rotation was intermittently interrupted by reciprocation cycles whenever the torque exceeded the preset threshold of 0.8 N·cm.

**Figure 1:**
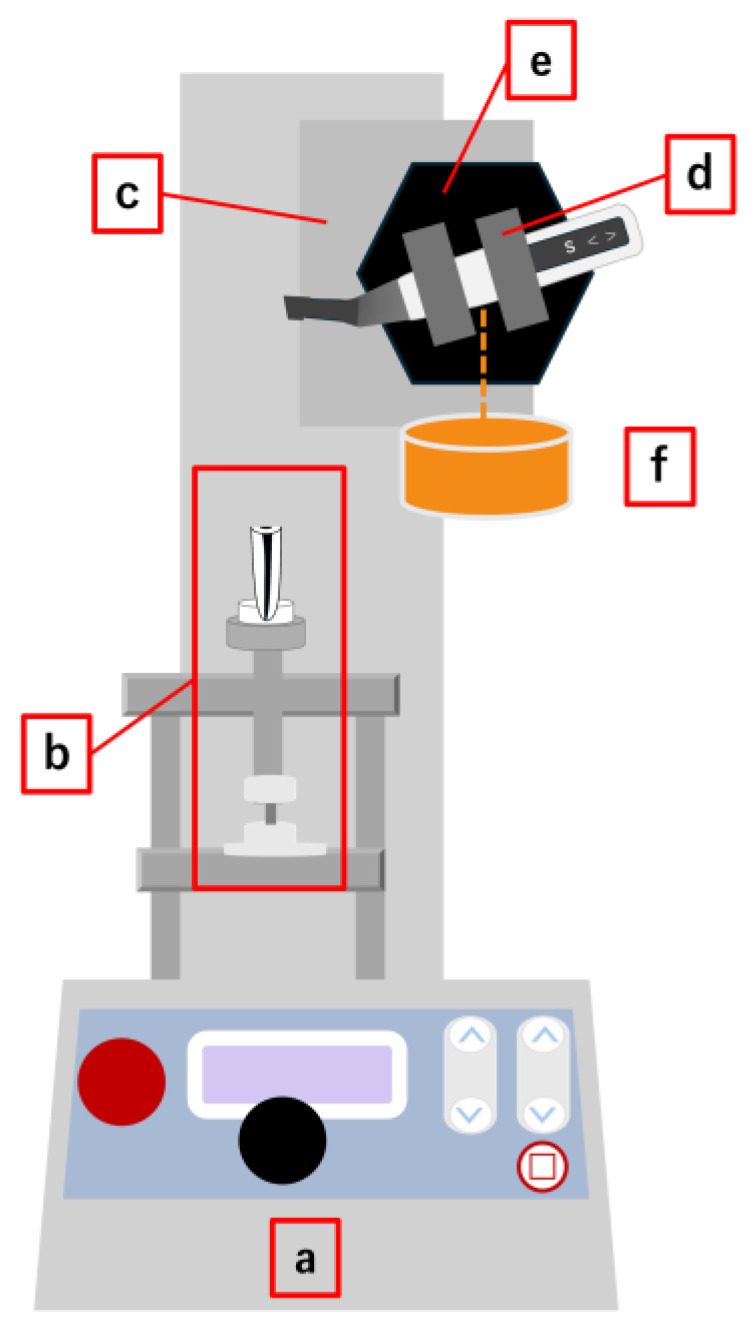
Schematics of the automated endodontic instrumentation device with controlled downward loading and torque/force sensing: (a) motorized testing apparatus, (b) torque/force sensing unit, (c) movable stage, (d) handpiece holder, (e) electromagnet, and (f) weight.

An automated endodontic instrumentation device with controlled downward loading and torque/force sensing (Figure 1) was used to ensure reproducibility, as described in prior studies [13,25]. Briefly, the instrumentation device comprised the Tri-Auto ZX motor mounted on a motorized testing apparatus (MX2-500N; Imada, Aichi, Japan). Specimens were fixed in a rigid holder connected to the torque/force sensing system. A handpiece holder, custom-made for this apparatus, was secured on the stand via an electromagnet and suspended with counterweights on pulleys to maintain balance. Activation of the electromagnet caused the handpiece and stage to advance simultaneously at 50 mm/min; upon deactivation, the handpiece was allowed to drop under applied loads of 1, 2, or 3 N, corresponding to the selected weights.

The handpiece was set to perform one of the following three motion patterns depending on the clockwise torque level detected by the motor.

Movement 1, initial insertion: if the torque remained under 0.2 N·cm, activation of the electromagnet caused the handpiece and stage to descend for 2 s, followed by an upward motion for 1 s, both at a rate of 50 mm/min.

Movement 2, free descent: if the torque was between 0.2 and 2.5 N·cm, the electromagnet was turned off, and the handpiece was released to move freely under predetermined loads of 1, 2, or 3 N.

Movement 3, withdrawal and reset: once the torque surpassed 2.5 N·cm, the electromagnet was activated, resulting in an upward displacement of the handpiece for 3 s at a speed of 50 mm/min.

Torque and force were measured using a sensing system comprising strain gauges (KFG-2-120-D31-11, Kyowa, Tokyo, Japan) for torque detection and a load cell (LUX-B-ID, Kyowa) for vertical force measurement [13,25]. Signals were amplified using a PCD-400A amplifier (Kyowa) and recorded using DCS-100A data acquisition software, version 04.31 (Kyowa). During instrumentation, both clockwise and counterclockwise torque, as well as apical and coronal vertical forces, were continuously monitored, and the maximum values were determined for each parameter.

To ensure accurate measurement during instrumentation, the strain gauges and load cell used in this study were factory-calibrated before the experiments. Calibration certificates confirmed the linearity (0.59–0.68% of the rated output), hysteresis, rated output, and calibration constants for clockwise and counterclockwise torque measurements.

### 2.4. Root Canal Instrumentation

The 54 extracted mandibular premolars were assigned to six groups (*n* = 9 each), namely CR-1N, CR-2N, CR-3N, OTR-1N, OTR-2N, and OTR-3N, based on the rotation and downward load. The downward load values (1–3 N) were selected based on previously published protocols [13,25] and preliminary testing, which showed that higher loads frequently caused instrument fracture and reduced experimental reproducibility.

For each tooth, glide path preparation was performed using the JIZAI Pre 013 NiTi rotary glide path instrument (MANI, Utsunomiya, Japan). Patency and working length were confirmed in all root canals, both before and after the shaping procedure.

Root canals were instrumented using JIZAI NiTi rotary instruments (MANI) with the aforementioned load-controlled device, employing the following sequence: I (#25, 0.04 taper), II (#25, 0.06 taper), and III (#35, 0.04 taper), with each instrument reaching the established working length. During instrumentation, 3% sodium hypochlorite (Dental Antiformin, Nippon Shika Yakuhin, Shimonoseki, Japan) was used to fill the canal, and RC-Prep (Premier, Philadelphia, PA, USA) served as a lubricant. The canals were irrigated with 3.0 mL of 17% EDTA (MD Cleanser, Meta Biomed, Cheongju, Republic of Korea) and 3.0 mL of 3% sodium hypochlorite at each instrument change. Each instrument was used for the preparation of only one canal.

The working time was measured for each extracted tooth using a stopwatch, considering the total sum of the individual instrumentation times for I, II, and III.

### 2.5. Canal-Centering Ratio Determination

Each specimen was precisely repositioned on the customized cylindrical jig and rescanned using the same micro-CT settings described in Section 2.2; the images were then processed using Amira software (v. 2023.2; Thermo Fisher Scientific, Waltham, MA, USA). After superimposing the pre- and post-instrumentation scans, a reference line was defined as the shortest path connecting the mesial root outline to the mesial boundary of the post-instrumentation canal, followed by the extension of this line to the distal root boundary. Measuring points corresponding to the mesial and distal canal outlines, both before and after instrumentation, were plotted onto the reference line. Canal-centering ratios were assessed at 1, 3, and 5 mm from the apex. The distances between the pre- and post-instrumentation outlines were measured for each side (X1 for mesial, X2 for distal), and the canal diameter after instrumentation (Y) was also recorded (Figure S1). The canal-centering ratio was calculated using the following formula: (X1 − X2)/Y [26]. A value of 0 corresponds to perfect centering.

### 2.6. Statistical Analysis

Statistical analysis was carried out using SPSS software (v22.0; IBM, Armonk, NY, USA). Normality of the data was assessed using the Shapiro–Wilk test, and homogeneity of variance was checked using Levene’s test. A two-way analysis of variance, followed by Tukey’s post hoc test, was performed to evaluate the torque, force, instrumentation time, and canal-centering ratio. A *p*-value < 0.05 indicated a significant difference.

## 3. Results

**Figure 2:**
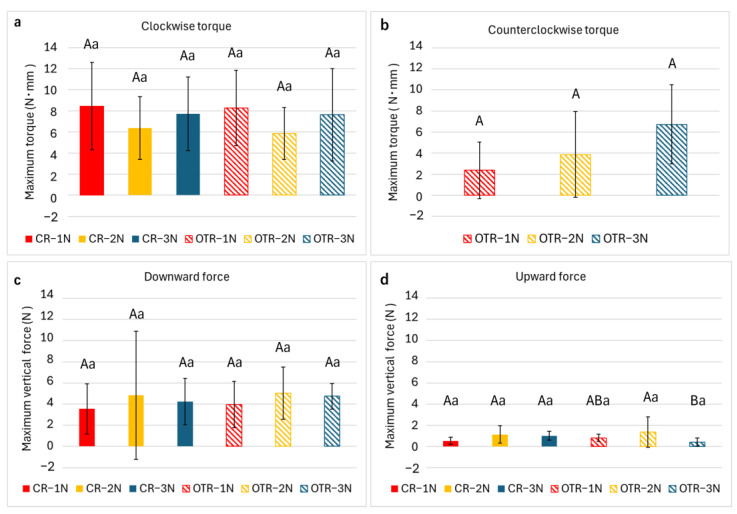
Maximum values of (**a**) clockwise torque, (**b**) counterclockwise torque, (**c**) downward vertical force, and (**d**) upward vertical force measured during automated root canal instrumentation. Results are expressed as mean ± standard deviation (*n* = 9). Different uppercase letters denote statistically significant differences (*p* < 0.05) within the same rotational mode. Different lowercase letters denote statistically significant differences (*p* < 0.05) within the same downward load condition.

All specimens were successfully instrumented with no instrument fracture or ledging observed. No significant differences were observed across all groups regarding clockwise torque, counterclockwise torque, or downward vertical force (Figure 2a–c). In CR mode, only a clockwise torque was generated. For the upward vertical force, different rotational modes did not show significant variation (Figure 2d). Among the OTR groups, OTR-3N produced a significantly lower upward force than OTR-2N (*p* < 0.05, Figure 2d).

**Figure 3:**
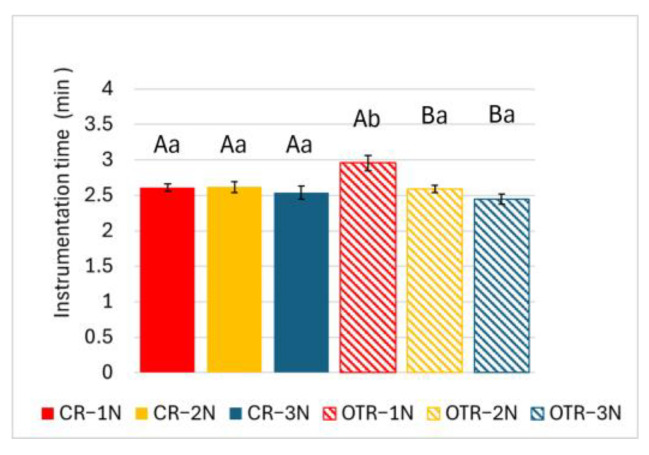
Instrumentation time for continuous rotation (CR) and optimum torque reverse (OTR) motion. Results are expressed as mean ± standard deviation (*n* = 9). Different uppercase letters denote statistically significant differences (*p* < 0.05) within the same rotational mode. Different lowercase letters denote statistically significant differences (*p* < 0.05) within the same downward load condition.

As presented in Figure 3, OTR-1N took significantly more time than CR-1N. Among the OTR groups, OTR-1N took significantly longer than both OTR-2N and OTR-3N (*p* < 0.05).

**Figure 4:**
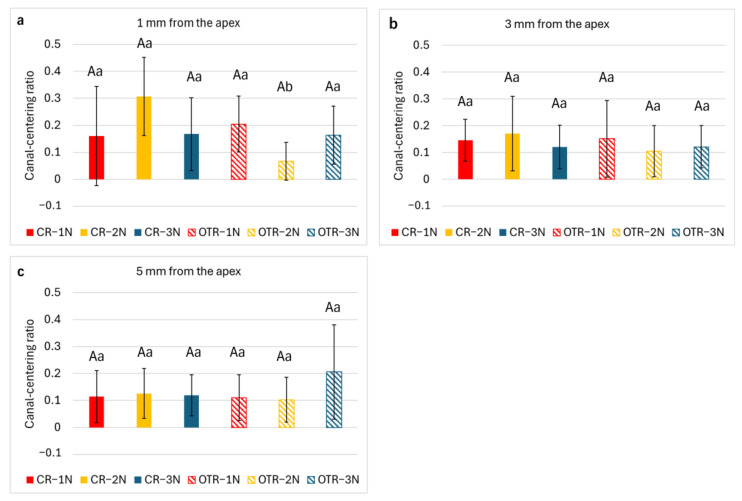
Canal-centering ratio at 1, 3, and 5 mm from the apex. (**a**) 1 mm from the apex; (**b**) 3 mm from the apex; (**c**) 5 mm from the apex. Results are expressed as mean ± standard deviation (*n* = 9). Different uppercase letters denote statistically significant differences (*p* < 0.05) within the same rotational mode. Different lowercase letters denote statistically significant differences (*p* < 0.05) within the same downward load condition.

As shown in Figure 4, CR-2N demonstrated a significantly higher canal-centering ratio compared with OTR-2N at 1 mm from the apex (*p* < 0.05). At 3 and 5 mm from the apex, no significant differences were detected between the groups.

## 4. Discussion

To our knowledge, this study is the first effort to analyze the relationship between the rotational mode and the magnitude of the downward load used during NiTi instrumentation. The findings led to a partial rejection of the null hypothesis. Specifically, significant differences were observed in the canal-centering ratio between CR-2N and OTR-2N at 1 mm from the apex, and in the instrumentation time between CR-1N and OTR-1N.

JIZAI I, II, and III (MANI) were selected as a representative example of contemporary heat-treated NiTi rotary instruments. JIZAI is a constant-tapered NiTi rotary instrument with shape memory properties resulting from the heat treatment. It has an off-centered quasi-rectangular cross-section with a radial land along one short edge and was designed to reduce screw-in forces while facilitating debris removal [27]. According to the manufacturer, JIZAI instruments are recommended for use in both CR mode (500 rpm, torque limit of 3.0 N·cm) and OTR mode (500 rpm, torque limit of 1.0 N·cm) [27]. Whereas our previous studies [13,25] evaluated only the CR mode, the present study used an instrument compatible with both CR and OTR to examine the effects of rotational mode.

In NiTi rotary root canal instrumentation, torque and downward vertical force play critical roles in procedural safety. When the downward force becomes excessive, the instrument may tightly bind against the canal wall, thereby increasing the torque and raising the risk of instrument fracture [28]. Previous studies have shown that a higher downward load produces a corresponding increase in the vertical and screw-in forces [13,25]. Thus, one major concern with applying a higher downward load is the potential increase in mechanical stress on NiTi instruments, which can lead to instrument fracture or excessive force on the root canal wall [29]. Importantly, increasing the downward load from 1 to 3 N did not increase clockwise torque, counterclockwise torque, or downward vertical force in our study. This result may seem unexpected, but it can be explained by the design of the JIZAI instrument. Because this instrument engages the canal wall only moderately—partly owing to its radial landed design—the cutting resistance may not reach levels that cause additional mechanical stress. Under these conditions, a higher downward load can improve shaping efficiency and canal-centering ability without producing the excessive torque that is often considered a risk. Consequently, our findings suggest that, within the tested range, using JIZAI instruments (MANI) with a greater downward load can improve cutting efficiency without proportionally increasing mechanical stress on the instruments.

Additionally, an excessive screw-in force may induce sudden instrument engagement within the root canal, resulting in sharp torsional stress and an increased risk of separation [9,14]. The screw-in force generally increases with higher rotational speed [12] and is influenced by operator-related factors, including the magnitude of the downward load [13,25] and the speed [12] and amplitude [14] of the pecking motion. OTR is expected to decrease the screw-in force [18]. In this study, the OTR-3N demonstrated a significantly lower screw-in force than OTR-2N, indicating that higher downward loads can help suppress a screw-in tendency. Thus, in addition to maintaining stable torque values, increasing the downward load was associated with a reduction in the screw-in force—a favorable outcome—among the OTR groups. This reduction in screw-in force may reflect the activation of multiple reciprocation cycles under higher downward load, which intermittently counteracted the file’s apical pulling tendency in the narrow canal.

These findings are in agreement with an earlier report [25] showing that applying moderate downward loads during instrumentation with flexible, heat-treated NiTi instruments does not increase the screw-in force. This phenomenon may be explained by the enhanced cutting efficiency achieved under an adequate downward load; the instrument cuts the dentin more effectively, resulting in smoother apical progression and reduced binding that would otherwise generate screw-in forces. However, the frequency and cumulative duration of OTR reciprocation, as well as the percentage of instrumentation time spent in reciprocation, were not quantified. Time-resolved analysis may be needed to clarify their contribution to the suppression of screw-in force.

Despite the kinematic differences between CR and OTR modes, both produced comparable levels of mechanical stress during instrumentation. For all downward loads, no significant differences were observed between the two rotational modes in any of the measured torque or force parameters. These results appear to contradict a previous report [18], which concluded that OTR reduces torque and force. However, this discrepancy may be attributed to differences in the applied downward load during root canal instrumentation, as highlighted in another study [13]. In the present study, applying a carefully controlled, appropriate downward load likely prevented the generation of excessive torque, even under CR mode. Consequently, the primary advantage of OTR—automatically switching to reciprocation upon torque exceedance—may not have been fully employed or statistically discernible. These findings suggest that the mechanical stress generated during canal shaping is governed more by the applied vertical force than by the kinematic motion. Accordingly, the OTR mode can be used under loading conditions comparable to those in CR without imposing additional stress on either the instrument or the root canal structure.

Canal-centering ratios were evaluated using two-dimensional cross-sectional analysis. Although three-dimensional volumetric analysis provides a more comprehensive depiction of canal morphology, the two-dimensional approach was chosen to enable standardized, reproducible assessment at clinically relevant levels and to reduce the impact of anatomical variability among extracted human teeth. The measurement at 1 mm from the apex is particularly relevant for clinical applications because this apical region is critical for achieving an adequate apical seal and is most susceptible to procedural errors [30,31,32].

The rotational mode significantly influenced canal shaping quality at the apical level, although the torque and force generation were equivalent. At 1 mm from the apex, OTR-2N showed a significantly lower canal-centering ratio than CR-2N. Even though the effect was confined to a localized region, it warrants attention given the importance of precise apical preparation. This limited but noteworthy observation differs from earlier studies reporting comparable centering ability between reciprocation and continuous rotation [33]. This indicates that OTR’s primary advantage—brief, partial disengagement of the instrument through torque-sensing reverse motion—is critical in preventing excessive “screwing-in” and in optimizing the instrument’s angle of engagement with the canal wall. Furthermore, while the superiority of NiTi alloys over stainless steel in maintaining canal morphology is well established [26], the present findings demonstrate that the motion pattern is a crucial independent factor that influences the maintenance of the original anatomy, particularly at the critical apical level. This enhanced apical centering ability may be related to the intermittent reciprocating motion, which likely reduces continuous cutting contact with the outer canal wall.

The OTR-1N group required significantly longer instrumentation time than the CR-1N group, indicating that, in addition to affecting shaping quality, rotational mode significantly influenced instrumentation efficiency, particularly at lower downward loads. In contrast, the three CR groups exhibited no significant differences. These findings indicate that OTR mode exhibits reduced cutting efficiency when insufficient downward load is applied, likely due to the frequent activation of reciprocating motion, which interrupts continuous cutting action [34]. When an adequate downward load is applied, the frequency of OTR activation appears to decrease, achieving a cutting efficiency comparable to that observed in CR mode.

One advantage of this study was the use of extracted human teeth, thereby replicating the clinical conditions more accurately than previous studies using simulated resin canals. Resin blocks do not replicate the mechanical properties of dentin, including its microhardness (approximately 35–40 kg/mm^2^) [15], moisture content, and tubular structure. Considering that NiTi instruments are primarily designed for shaping curved canals, and that instrument fracture is more likely to occur in curved root canals, moderately curved mandibular premolars (15–25°) were chosen for this study. Moreover, anatomical matching was carried out using three parameters—Schneider’s angle [23], and the canal cross-sectional areas at 1 and 5 mm from the apex—based on preoperative micro-CT scans. Although anatomical variability is an inherent limitation of extracted teeth, rigorous matching helped ensure comparable baseline conditions across groups, thus minimizing variability.

Although this study demonstrated that OTR mode with a moderate downward load offers advantages for root canal instrumentation, several limitations must be considered. The curvature range tested (15–25°) represents commonly encountered anatomies. However, it excludes severely curved canals where instrument stress and fracture risk are higher, limiting generalizability to more complex cases. The only significant difference in canal-centering ability was observed at 1 mm from the apex under the 2 N condition. The inconsistent findings across other levels may have been influenced by anatomical variation or other uncontrolled factors. The torque values in the OTR groups may have been influenced by the relatively low torque threshold (0.8 N·cm), which triggers early reciprocation and limits torque buildup. While these lower torque values may partly reflect the protective nature of the OTR and should be interpreted as clinically meaningful, they could complicate direct comparisons with CR (3 N·cm). Moreover, because only one NiTi rotary system (JIZAI) was assessed, it remains unclear whether the observed effects are system-specific to its design and metallurgy or applicable to other systems. Further studies involving multiple systems are needed to determine the generalizability of these findings. Finally, several clinical factors are not represented in this experimental model, such as individual operator skills, tooth angulation within the oral cavity, and mouth opening limitations. Therefore, careful consideration is required for direct clinical application.

From a clinical perspective, this study provides important implications for selecting appropriate rotational modes and downward loads during root canal instrumentation. In CR mode, operators can apply a downward load of 1–3 N without markedly affecting performance. However, when using OTR mode, applying a moderate downward load of approximately 3 N is advisable. This recommendation is supported by multiple observations. OTR-1N showed significantly longer instrumentation times, whereas OTR-2N achieved better apical centering while maintaining efficient working times. Additionally, OTR-3N exhibited significantly lower screw-in forces than OTR-2N, indicating that a moderate downward load offers an optimum balance among efficient cutting, minimized canal deviation, and reduced stress on the root structure.

## 5. Conclusions

Within the limitations of this study—namely the use of moderately curved canals and a single heat-treated NiTi system (JIZAI)—OTR mode with a downward load of 2–3 N provided root canal shaping efficiency comparable to CR while controlling the screw-in force and resulting in a smaller canal-centering ratio in the apical region. These findings indicate that OTR can be considered a promising alternative to CR for preparing curved canals with heat-treated NiTi instruments.

## Figures and Tables

**Table 1:** Morphometric values of root canals before root canal instrumentation.

Canal Cross-Sectional Area	Continuous Rotation Mode			Optimal Torque Reverse Mode			*p*
	1 N	2 N	3 N	1 N	2 N	3 N	
1 mm from the apex (mm^2^)	0.021 ±0.008	0.020 ±0.008	0.020 ±0.008	0.020 ±0.008	0.020 ±0.006	0.020 ±0.007	1.000
5 mm from the apex (mm^2^)	0.058 ±0.014	0.074 ±0.037	0.066 ±0.017	0.072 ±0.028	0.057 ±0.025	0.048 ±0.017	0.254
Canal curvature (degree)	20.5 ±3.69	17.7 ±2.48	19.6 ±4.45	17.8 ±3.42	17.8 ±3.42	17.8 ±2.48	0.392

Values are expressed as the mean and SD (*n* = 9). The *p*-values were obtained from one-way analysis of variance.

## Data Availability

The original contributions presented in this study are included in the article/Supplementary Material. Further inquiries can be directed to the corresponding author.

## Supplementary Materials

The following supporting information can be downloaded at https://www.mdpi.com/article/10.3390/ma19010108/s1. Figure S1: Representative superimposed cross-sectional image showing the reference line and measuring points used to calculate the canal-centering ratio. X1 and X2 represent the mesial and distal distances, respectively, between the canal outlines before and after instrumentation (orange lines). Y indicates the canal diameter after instrumentation (green line). The blue line represents the reference line used to determine the measuring points.

## References

[1] Srivastava S. (2024). Root canal instrumentation: Current trends and future perspectives. Cureus.

[2] Gomes B.P.F.A., Aveiro E., Kishen A. (2023). Irrigants and irrigation activation systems in Endodontics. Braz. Dent. J..

[3] Tomson P.L., Adams N., Kavanagh D., Virde S.S. (2025). Non-surgical endodontics: Contemporary biomechanical preparation of the root canal system. Br. Dent. J..

[4] Hou X., Yahata Y., Hayashi Y., Ebihara A., Hanawa T., Suda H. (2011). Phase transformation behaviour and bending property of twisted nickel-titanium endodontic instruments. Int. Endod. J..

[5] Gomes M.S., Vieira R.M., Böttcher D.E., Plotino G., Celeste R.K., Rossi-Fedele G. (2021). Clinical fracture incidence of rotary and reciprocating NiTi files: A systematic review and meta-regression. Aust. Endod. J..

[6] Hu W., Whitten B., Sedgley C., Svec T. (2014). Effect of three NiTi files on transportation of the apical foramen. Int. Endod. J..

[7] Berutti E., Chiandussi G., Paolino S.D., Scotti N., Cantatore G., Castellucci A. (2012). Damiano Pasqualini Canal shaping with WaveOne Primary reciprocating files and ProTaper system: A comparative study. J. Endod..

[8] Zupanc J., Vahdat-Pajouh N., Schäfer E. (2018). New thermomechanically treated NiTi alloys—A review. Int. Endod. J..

[9] Ha J.H., Cheung G.S.P., Versluis A., Lee C.J., Kwak S.W., Kim H.C. (2015). ‘Screw-in’ tendency of rotary nickel-titanium files due to design geometry. Int. Endod. J..

[10] Liang Y., Yue L. (2022). Evolution and development: Engine-driven endodontic rotary nickel-titanium instruments. Int. J. Oral. Sci..

[11] Oh S., Seo J., Lee J., Kim H., Jang J., Chang S.W. (2022). Evaluation of design, mechanical properties, and torque/force generation of heat-treated NiTi glide path instruments. BMC Oral Health.

[12] Maki K., Ebihara A., Kimura S., Nishijo M., Tokita D., Okiji T. (2019). Effect of different speeds of up-and-down motion on canal centering ability and vertical force and torque generation of nickel-titanium rotary instruments. J. Endod..

[13] Maki K., Ebihara A., Unno H., Omori S., Nakatsukasa T., Kimura S., Okiji T. (2022). Effect of different downward loads on canal centering ability, vertical force, and torque generation during nickel-titanium rotary instrumentation. Materials.

[14] Ha J.H., Kwak S.W., Sigurdsson A., Chang S.W., Kim S.K., Kim H. (2017). Stress generation during pecking motion of rotary nickel-titanium instruments with different pecking depth. J. Endod..

[15] Hülsmann M. (2022). A critical appraisal of research methods and experimental models for studies on root canal preparation. Int. Endod. J..

[16] Yared G. (2008). Canal preparation using only one Ni-Ti rotary instrument: Preliminary observations. Int. Endod. J..

[17] Ahn S.Y., Kim H.C., Kim E. (2016). Kinematic effects of nickel-titanium instruments with reciprocating or continuous rotation motion: A systematic review of in vitro studies. J. Endod..

[18] Kimura S., Ebihara A., Maki K., Nishijo M., Tokita D., Okiji T. (2020). Effect of optimum torque reverse motion on torque and force generation during root canal instrumentation with crown-down and single-length techniques. J. Endod..

[19] Omori S., Ebihara A., Hirano K., Kasuga Y., Unno H., Nakatsukasa T., Kimura S., Maki K., Hanawa T., Okiji T. (2022). Effect of rotational modes on torque/force generation and canal centering ability during rotary root canal instrumentation with differently heat-treated nickel-titanium instruments. Materials.

[20] Tokita D., Ebihara A., Nishijo M., Miyara K., Okiji T. (2017). Dynamic torque and vertical force analysis during nickel-titanium rotary root canal preparation with different modes of reciprocal rotation. J. Endod..

[21] Pedullà E., Corsentino G., Ambu E., Rovai F., Campedelli F., Rapisarda S., Rosa G.R.L., Rapisarda E., Grandini S. (2018). Influence of continuous rotation or reciprocation of Optimum Torque Reverse motion on cyclic fatigue resistance of nickel-titanium rotary instruments. Int. Endod. J..

[22] (2004). Dentistry—Number Coding System for Rotary Instruments—Part 1: General Characteristics.

[23] Schneider S.W. (1971). A comparison of canal preparations in straight and curved root canals. Oral Surg. Oral Med. Oral Pathol..

[24] Kyaw M.S., Ebihara A., Kasuga Y., Maki K., Kimura S., Htun P.H., Nakatsukasa T., Okiji T. (2021). Influence of rotational speed on torque/force generation and shaping ability during root canal instrumentation of extracted teeth with continuous rotation and optimum torque reverse motion. Int. Endod. J..

[25] Maki K., Ebihara A., Luo Y., Kasuga Y., Unno H., Omori S., Kimura S., Okiji T. (2025). Thermal treatment prevents effects of downward loads on the screw-in force generation and canal-centering ability of nickel-titanium rotary instruments. Materials.

[26] Paleker F., van der Vyver P.J. (2016). Comparison of canal transportation and centering ability of K-files, ProGlider File, and G-Files: A micro-computed tomography study of curved root canals. J. Endod..

[27] Nakatsukasa T., Ebihara A., Kimura S., Maki K., Nishijo M., Tokita D., Okiji T. (2021). Comparative evaluation of mechanical properties and shaping performance of heat-treated nickel titanium rotary instruments used in the single-length technique. Dent. Mater. J..

[28] Schäfer E., Bürklein S., Donnermeyer D. (2022). A critical analysis of research methods and experimental models to study the physical properties of NiTi instruments and their fracture characteristics. Int. Endod. J..

[29] Moreinos D., Dakar A., Stone N.J., Moshonov J. (2016). Evaluation of time to fracture and vertical forces applied by a novel Gentlefile system for root canal preparation in simulated root canals. J. Endod..

[30] Chaniotis A., Ordinola-Zapata R. (2022). Present status and future directions: Management of curved and calcified root canals. Int. Endod. J..

[31] Gulabivala K., Ng Y.L. (2023). Factors that affect the outcomes of root canal treatment and retreatment—A reframing of the principles. Int. Endod. J..

[32] Valverde Haro H.P., Rupaya C.R.G., Alves F.R.F. (2024). Procedural errors detected by cone beam tomography in cases with indication for retreatment: In vivo cross-sectional study. Restor. Dent. Endod..

[33] Naseri M., Paymanpour P., Kangarloo A., Haddadpur S., Dianat O., Ketabi M.A. (2016). Influence of motion pattern on apical transportation and centering ability of WaveOne single-file technique in curved root canals. Dent. Res. J..

[34] Tantiwanichpun B., Kulvitit S. (2023). Efficiency and complications in root canal retreatment using nickel titanium rotary file with continuous rotation, reciprocating, or adaptive motion in curved root canals: A laboratory investigation. BMC Oral Health.

