# Supplementary figures and images for: Impact of Downward Load and Rotational Kinematics on Root Canal Instrumentation with a Heat-Treated Nickel–Titanium Rotary Instrument
Source: Materials (Basel). 2025 Dec 28;19(1):108. doi: 10.3390/ma19010108 (PMC12786967; doi:10.3390/ma19010108)

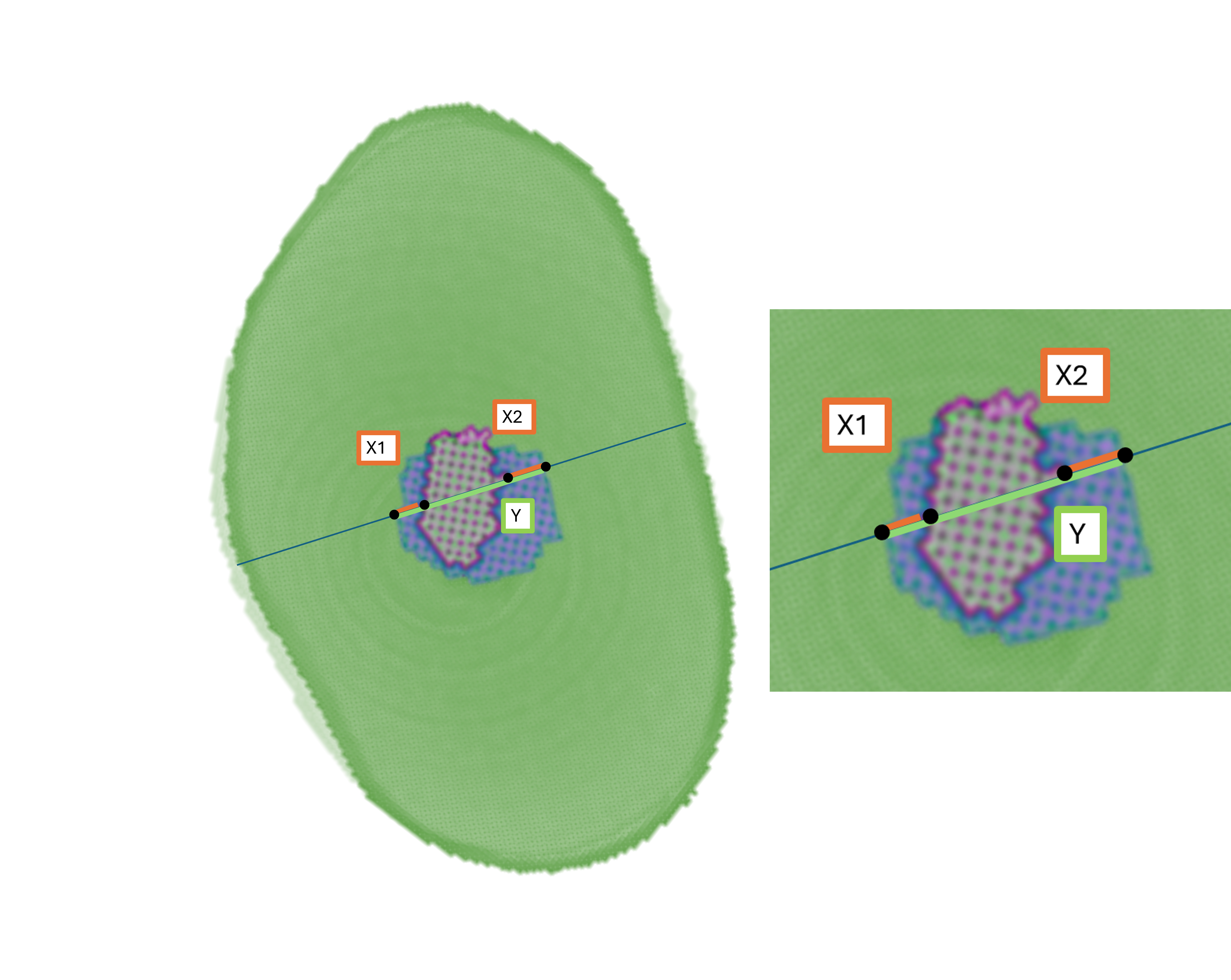

Supplement: Supplementary file 1 [file materials-19-00108-s001.zip › S1.tif]
